# Optimizing integrated imaging service delivery by tier in low-resource health systems

**DOI:** 10.1186/s13244-021-01073-8

**Published:** 2021-09-16

**Authors:** Kristen DeStigter, Kara-Lee Pool, Abimbola Leslie, Sarwat Hussain, Bien Soo Tan, Lluis Donoso-Bach, Savvas Andronikou

**Affiliations:** 1 Department of Radiology, Larner College of Medicine, University of Vermont, 111 Colchester Avenue Main Campus, McClure, Level 1, Burlington, VT 05401 USA; 2 RAD-AID International, 8004 Ellingson Drive, Chevy Chase, MD 20815 USA; 3 Department of Radiology, University of Massachusetts, 55 North Lake Ave, Worcester, MA 01655 USA; 4 Department of Vascular and Interventional Radiology, Singapore General Hospital, Outram Rd, Singapore, 169608 Singapore; 5 Department of Medical Imaging, Hospital Clínic of Barcelona, University of Barcelona, C. de Villarroel, 170, 08036 Barcelona, Spain; 6 Department of Pediatric Radiology, The Children’s Hospital of Philadelphia and the Perelman School of Medicine, University of Pennsylvania, Philadelphia, USA

**Keywords:** Global health, Radiology, Population health, Diagnostic imaging

## Abstract

Access to imaging diagnostics has been shown to result in accurate treatment, management, and optimal outcomes. Particularly in low-income and low-middle-income countries (LICs, LMICs), access is limited due to a lack of adequate resources. To achieve Sustainable Development Goal (SDG) 3, access to imaging services is critical at every tier of the health system. Optimizing imaging services in low-resource settings is best accomplished by prescriptive, integrated, and coordinated tiered service delivery that takes contextual factors into consideration. To our knowledge, this is the first recommendation for optimized, specific imaging care delivery by tier. A model for tier-based essential imaging services informs and guides policymakers as they set priorities and make budgetary decisions. In this paper, we recommend a framework for tiered imaging services essential to reduce the global burden of disease and attain universal health coverage (UHC). A lack of access to basic imaging services, even at the lowest tier of the health system, can no longer be justified by cost. Worldwide, affordable modalities of modern ultrasound and X-ray are becoming an accessible mainstay for the investigation of common conditions such as pregnancy, pneumonia, and fractures, and are safely performed and interpreted by qualified professionals. Finally, given the vast gap in access to imaging resources between LMICs and high-income countries (HICs), a scale-up of tiered imaging services in low-resource settings has the potential to reduce health disparities between, and within countries. As the access to appropriately integrated imaging services improves, UHC may be achieved.

## Key points

- Access to imaging diagnostics has been shown to result in accurate treatment, management, and optimal outcomes.
- Access to imaging diagnostics in LMICs is limited due to lack of adequate resources.
- Tiered imaging services in low-resource settings have the potential to reduce health disparities between, and within countries, and may be implemented according to local context and setting.
- Investment in strengthening national policy around essential imaging diagnostics for tiered service delivery, with a focus on technology, human resources, infrastructure development, and quality management, will support primary care and specialty services for a healthy population.

## Introduction

Imaging is an essential component of health systems where image-based diagnosis and intervention play a central role in individual patient care, through screening, diagnosis, treatment, and surveillance. Reliable and timely imaging results aid decision making for most health specialties and services, yet imaging services remain neglected or even invisible on the world stage. Lack of imaging diagnostics disproportionately impacts resource-limited areas that leaves millions of people without even basic reliable diagnostic imaging services. Sustainable Development Goals (SDGs) 3 has 13 targets to decrease the burden of disease, and even though imaging is an essential element of disease diagnosis, management, and eradication, none of the 26 key performance indicators mentions imaging [1–4]. The WHO Universal Health Coverage for the year 2030 (UHC 2030) promises continued expansion of primary care, surgical and obstetric services to the world’s underserved regions, but says little about the imaging services required. The UHC 2030 jumps from preventive to curative, skipping the notion of “diagnosis” as a critical step [5]. The diagnostic gap for LICs and LMICs is more critical at the primary healthcare level, where access to even the simplest imaging is limited [6]. The health and economic benefits of investing in imaging services outweigh the cost, as calculated from future expenses avoided, with an estimated net survival benefit [4]. The disproportionately high burden of disease in low resource areas, especially sub-Saharan Africa [5], is worsened by a lack of access to affordable imaging technology, a limited skilled workforce, insufficient infrastructure, and limited processes for quality oversight, compounded by social inequities [6]. Many LMICs lack a placeholder for imaging services in their national health service strategic plans. A recent analysis of National Health Service Strategic Plans of all LICs and LMICs revealed that only 24% (19 countries) mentioned imaging /radiology services [7], which leaves imaging services unfunded, of low priority, and with little chance of operationalization.

In 2000, the UN Committee on Economic and Social Council (ECOSOC) described the right to the highest attainable standard of health, noting that health services should be available in adequate numbers, accessible, of good quality, and acceptable to all [8]. As a result of COVID-19, the UN Human Rights Council repeated that everyone, without exception, has the right to life-saving interventions [9]. Failure of the healthcare system to deliver services to low-income populations in LMICs has been attributed to lack of equity in health, demonstrating a need for fair distribution of health services, across all tiers of the healthcare system [10]. Tier-based imaging services addresses and promotes equity in access to basic imaging services.

The WHO Essential Diagnostic List (EDL) is a package of services in a pyramidal, tier-based healthcare system, where a primary health center serves the local community and refers to a higher level of care where expanded services are offered. This serves as a general recommendation for countries based on contextual circumstances, including the level of poverty, social preferences, operational challenges, and differences in disease burden [11]. To date, imaging has not been included in WHO EDLs across tiers [12].

In this paper, the top 21 diseases/conditions forecasted for 2030 (in the Global Burden of Disease Foresight data) [5] are converted to indications for imaging and matched with the radiological services required for primary diagnosis, diagnosis of complications, treatment, and surveillance (Table 1) based on the American College of Radiology (ACR) Appropriateness Criteria. Notably, imaging diagnosis and/or intervention is required across the board, underscoring the need to improve community access to imaging services through a tier-based system [13].

**Table 1:** Intersection of Forecasted (2030) Global Burden of Disease—top 21 causes—with Imaging Indication

Global burden disease foresight 2030 list of #1–21 causes of years of life lost per 100,000 (both sexes, age-standardized) [5]	Screening with imaging	Imaging for primary diagnosis (directly or contributory)	Imaging for diagnosis of complications of disease	Imaging-guided treatment	Imaging surveillance
1. Cardiovascular Disease [13]		x	x	x	x
2. Neoplasm [12, 19]	x	x	x	x	x
3. Diarrhea/lower respiratory tract infection [13]		x	x		
4. Neonatal conditions [13]		x	x	x	x
5. Chronic non-communicable diseases [13]		x	x	x	x
6. HIV/AIDS/TB [13]	x	x	x	x	x
7. Unintentional injuries (falls, drowning, fire) [13]		x	x	x	x
8. Transport injuries [13]		x	x	x	x
9. Chronic respiratory disease [13]		x	x	x	x
10. Self-harm and violence [13]		x	x	x	
11. Neglected tropical diseases (NTDs), malaria		x	x	x	x
12. Other non-communicable diseases [13]		x	x	x	x
13. Neurological [13]		x	x	x	x
14. Cirrhosis [13]		x	x	x	x
15. Digestive diseases [13]		x	x	x	x
16. Mental diseases [13]		x	x	x	x
17. Other communicable (STDs) [13]			x		
18. Nutritional deficiencies			x	x	
19. War and disaster [13]		x	x	x	x
20. Maternal disorders [13]	x	x	x	x	x
21. Musculoskeletal [13]		x	x	x	x

**Fig. 1:**
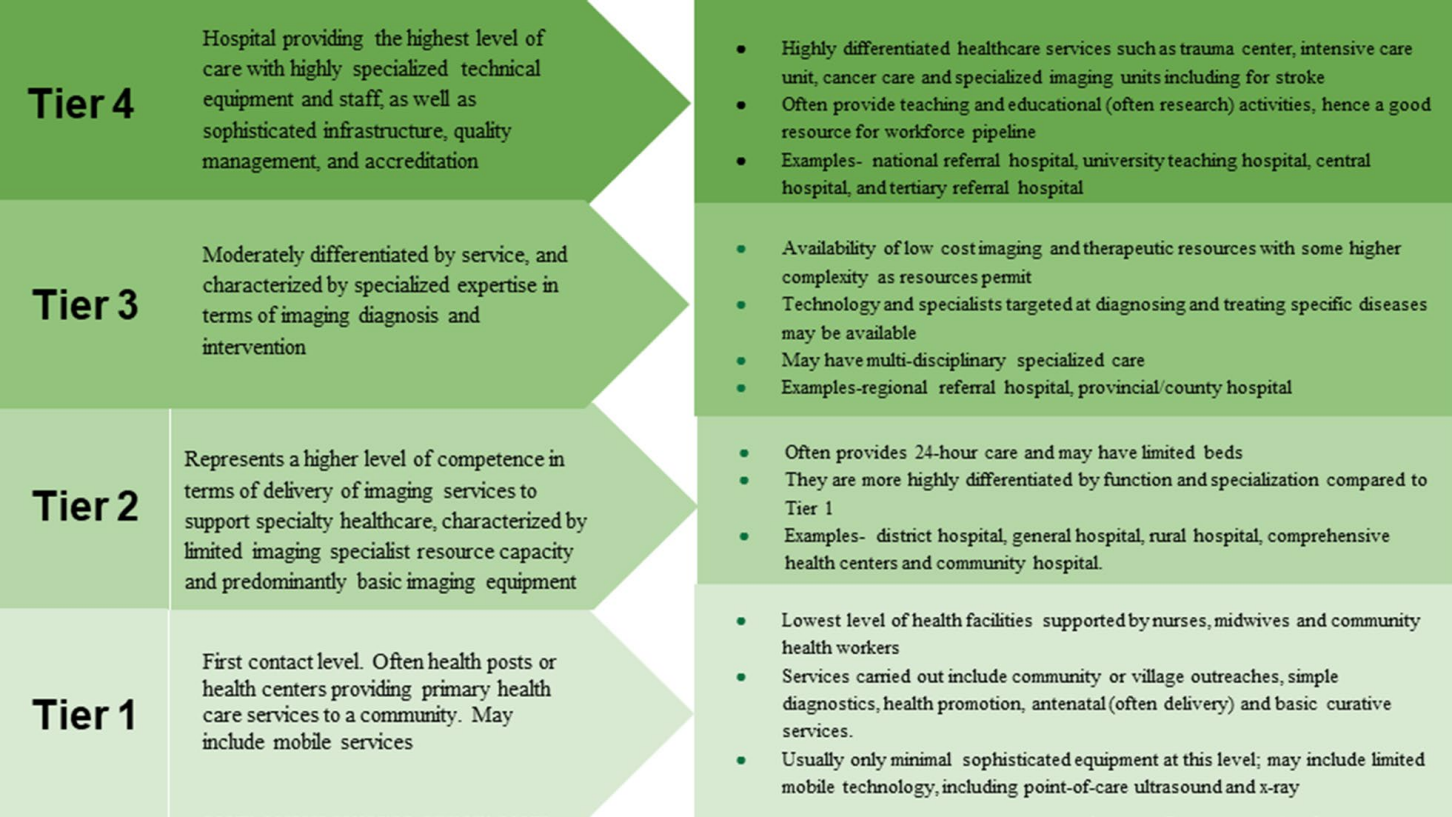
Tiers and levels of Health Systems. A darker color denotes a higher level of care with higher complexity and requirement for more specialized human and technological resources

Many countries, including LICs and LMICs, have pyramidal-based health systems with tiers of service, with anything from 3–6 tiers depending on the country, e.g., Zambia has three tiers [14]. Lower tiers of health systems usually lack the skills, facilities, or both to manage some illnesses and may be compelled to refer patients to higher tiers that are better equipped with the resources needed for appropriate care [15]. Differentiation of the diagnostic imaging services into tiers organizes features of gradually increasing complexity in the availability of basic to advanced diagnostic technologies, personnel offering specialized services, and /or advanced therapeutic technologies that facilitate diagnosis and treatment of complex conditions [16]. Imaging services are well-suited to four tiers (Fig. 1) where modern basic X-ray and ultrasound imaging is adaptable for a primary healthcare level and the most advanced services are offered at the highest level.

The following are the necessary components of integrated tiered imaging service delivery: *imaging technology, qualified human capacity, infrastructure (including information technology), and management of quality systems*. In addition, an established and functional pattern for referrals is essential, with patients able to access higher levels of care as needed. Specific guidelines may not fit all countries where contextual considerations prevail.

The goal of this paper is to provide a framework for optimizing integrated imaging delivery services by tier in low-resource health systems. Imaging diagnosis is a collaborative activity that requires multiple elements that all are fit for purpose: technology, workforce, infrastructure, and quality management.

## Technology

X-ray and ultrasound are considered traditional imaging modalities that yield the largest survival gains in low-income settings [17]. The WHO recommends that every woman should have at least one ultrasound during pregnancy [14]. Advanced imaging modalities such as computed tomography scan (CT), magnetic resonance imaging (MRI), and positron emission tomography (PET) are now standard practice in the diagnosis of many diseases. New applications enabling telemedicine and artificial intelligence for disease detection and workflow efficiency will continue to change the care of patients in all tiers.

Constraints for achieving access to all modalities at all levels of the health system include high costs and lack of availability of equipment, infrastructure, and skilled human resources [15]. New equipment is expensive, often compounded by taxes and duties. Often, all the costs of ownership are not considered, including physical space, siting, licenses, network, and consumables. Installation failures due to inconsistent power sources, poor network connectivity, and lack of qualified personnel are common [18]. Many sales in LMICs use third-party distributors for new and refurbished equipment, limiting application training for personnel and resulting in lower confidence to use the equipment. Additionally, many donated devices lack warranties, installation support, user manuals, and even available parts. These challenges affect technology ownership at all tiers of imaging service delivery but are least able to be addressed at the lowest tiers due to the requirement for skilled human resources. Often, radiologists and other imaging specialists are not consulted when imaging equipment is purchased even though adequate, and appropriate imaging technologies fit for purpose, are what empowers healthcare teams to provide optimal patient care. In addition, competing priorities become limiting factors to technology procurement, especially in LICs and LMICs.

In the integrated, tiered healthcare system the various imaging technology needs for each tier are determined based on imaging testing needs, resource constraints, population needs, and infrastructure requirements [16].

**Fig. 2:**
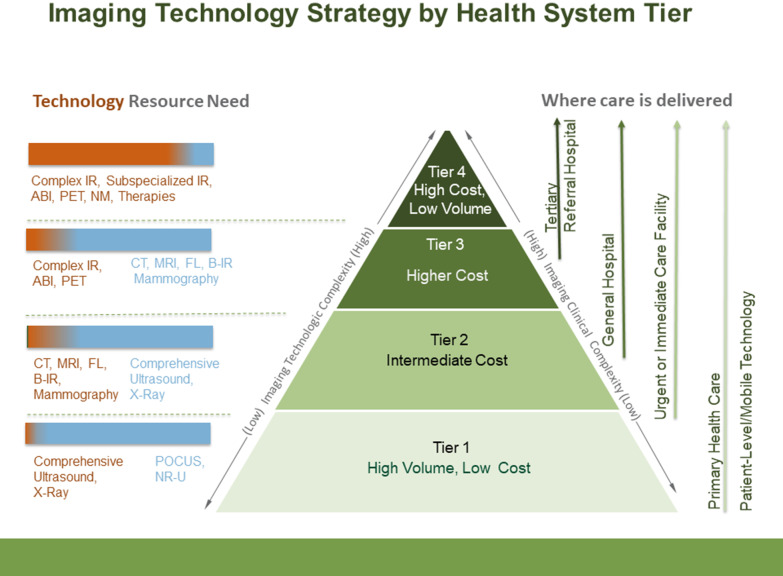
Recommended imaging technology strategy by health system tier. Higher levels on the pyramid denote increasing complexity. For each tier, red indicates a requirement for higher complexity of technology while blue indicates lower complexity of technology. (POCUS = Point of Care ultrasound; NR-U = non-radiologist ultrasound; CT = computed tomography; MRI = magnetic resonance imaging; FL = fluoroscopy; B-IR = BASIC Interventional Radiology; ABI = advanced breast imaging; PET = positron emission tomography; NM = nuclear medicine; subspecialized IR = neurointerventional)

**Fig. 3:**
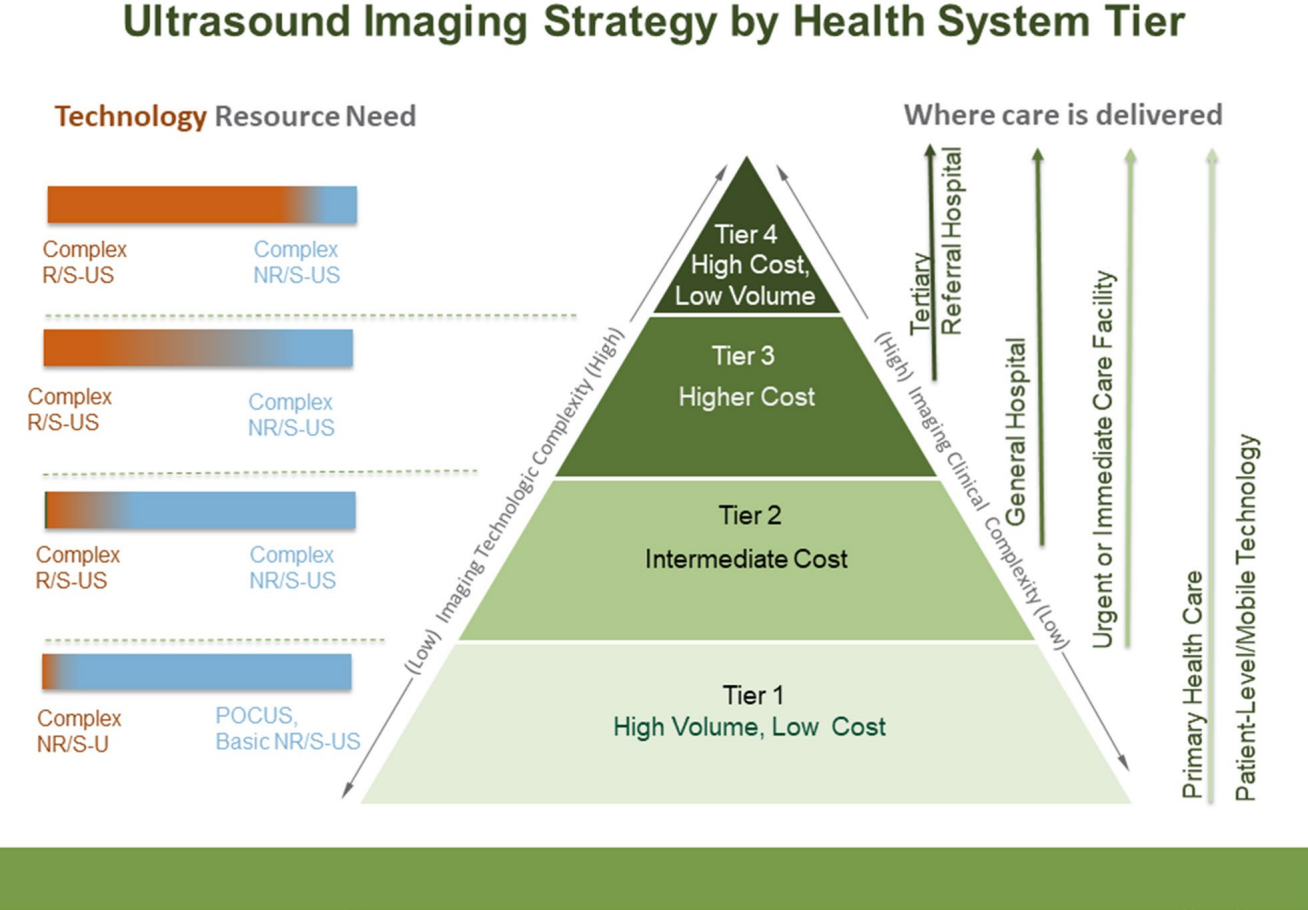
Recommended ultrasound imaging strategy by health system tier. Higher levels on the pyramid denote increasing complexity. For each tier, red indicates a requirement for higher specialization of service (technology and personnel), while blue indicates lower specialization requirements (POCUS = Point of Care ultrasound; NR/S-US = non-radiologist/non-specialist ultrasound; complex R/S-US = radiologist/specialist ultrasound)

For tier 1 in a limited resource environment, point-of-care ultrasound (POCUS) and X-ray should be available for reducing both underdiagnosis and overdiagnosis [17, 19] (Figs. 2 and 3). Basic ultrasound for obstetric care, for example, is a primary care requirement in Uganda [20]. POCUS requires no additional skilled personnel, requires a minimal power supply, is rugged for remote settings, and is relatively easy to teach and learn. It requires less infrastructure and training than other imaging modalities such as CT scans or MRIs. POCUS in a low-resource setting in rural Tanzania showed a change in diagnostic impression in over 40 percent of cases and helped providers identify new or additional diagnoses, improving patient care and outcomes [21]. Rapid point-of-care (POC) diagnostics are recommended by the WHO because they achieve the greatest impact on disease prevalence by improving the quality of care for screening programs and for individuals [22].

Tier 2 level facilities are usually the first level of referral and at the minimum should have comprehensive ultrasound plus X-ray and can include CT, MRI, fluoroscopy (FL), basic interventional radiology (B-IR), or even mammography, if resources (qualified staff and quality management) allow. Since clinical services offered at Tier 3 are more differentiated in terms of function and expertize, CT, MRI, FL, basic interventional radiology, and mammography services should be available at the minimum, and where more resources are available, complex interventional radiology with subspecialized protocols, advanced breast imaging (ABI), and PET could be added.

Tier 4 levels are the highest-level referral centers, often national referral and academic hospitals, that are tasked with providing the highest level of healthcare including complex IR, subspecialized IR (e.g., interventional neurology), ABI, PET, nuclear medicine (NM) and image-guided or image-directed therapies should be available, over and above Tier 3 services.

Availability of essential technology matching the most common indications for imaging at each tier can provide cost-effective measures through improved screening, diagnosis, treatment, and outcomes. For example, the scaling up of imaging services simulated for eleven cancer types has been shown to avert about 2·46 million deaths, accounting for 3·2% of worldwide deaths and 54·92 million life-years saved [4]. As a further example, Interventional Radiology (IR) could provide greater benefits for LICs and LMICs, relative to HICs, due to the high mortality rates associated with the surgical and anesthesia complications they experience in practice [23].

## Human resources

Human resources at each tier should be appropriate for the services provided. Radiologists being qualified medical doctors with additional specialty training in diagnostic imaging represent the highest tier of the diagnostic imaging workforce. Limiting factors for producing radiologists locally include the availability of medical graduates, infrastructure (equipment), and expertize (qualified radiologists) to provide training, usually in a four-to-five-year program.

**Fig. 4:**
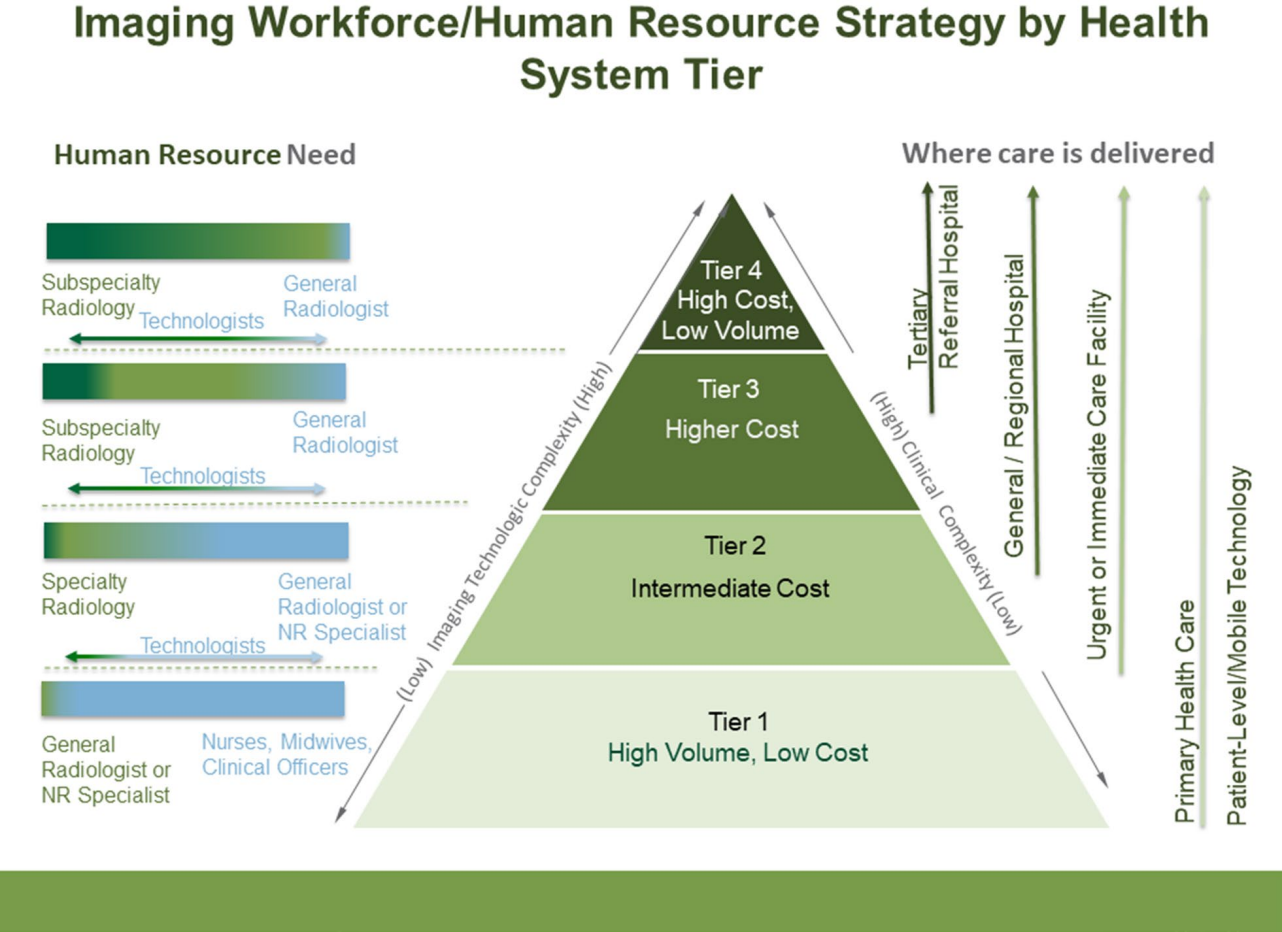
Recommended imaging workforce/human resource strategy by health system tier. Higher levels on the pyramid denote increasing complexity and requirement for specialization of staff. Green denotes a requirement for higher-level specialization or even sub-specialization (e.g., neurointerventional radiology, oncologic imaging), while blue denotes lower-level specialization. Technologists / radiographers are required at all tiers above primary care centers and may also have a role and primary care level even though not essential (NR = Non-Radiology; technologists = radiographic technologists, radiographers, sonographers)

General radiologists, accredited nationally as medical specialists, provide services in most fields of diagnostic imaging and some basic interventional radiology. They interpret diagnostic imaging with physician-level insight and should be available in tiers 2–4 where specialized radiologic equipment is available (Fig. 4). Radiologists can become subspecialized with further training, in areas such as neuroradiology, cardiothoracic imaging, abdominal imaging, breast imaging, pediatric imaging, musculoskeletal imaging, and vascular interventional radiology. Subspecialist radiologists are ideally suited for tier 4, a tertiary referral, and academic centers, but are a scarce resource in LICs and LMICs. Although not required in tier 1, general radiologists can play a role in performing primary-care level ultrasound and training POCUS practitioners.

Non-radiologists, including radiographers (X-ray technologists), sonographers, physicists, nurses, etc., are integral members of diagnostic imaging services and are required at all 4 tiers to provide the images. Formal training on modality applications is necessary for accreditation as a technologist/ sonographer, followed by regular in-service training and performance evaluation.

Established radiologist and technologist training programs exist in many LICs and LMICs but some countries lacking full radiologist training capabilities, send candidates to neighboring and distant countries for training.

Ideally, a sonographer (technologist) should staff tier 1 but imaging services can be offered (performed and interpreted) by training a non-radiologist/ non-technologist to perform some imaging through ‘task shifting,’ a strategy for addressing skilled healthcare worker shortages in developing countries [24, 25]. Task shifting is prevalent in African tier 1 facilities in Malawi, Uganda, and Zimbabwe where the benefits such as reduced waiting, faster turnaround times, and lower costs outweigh possible harms [25–29]. A global shortage of radiologists and increased demand for image interpretation has resulted in task-shifting to radiographers [30]. Primarily this relates to reporting appendicular X-rays from emergency departments [31] with evidence that radiographers are as accurate as radiologists [31–33] and more accurate than medical officers [34]. Implementation of teleradiology services and/or Artificial Intelligence (AI) can augment task shifting in tier 1 facilities. Radiographer extenders/advanced practice providers are well established in the United Kingdom [35–38] at all tiers of service but do not replace radiologists in tiers 2–4.

One notable form of task-shifting is the performance of point-of-care ultrasound (POCUS) by non-radiologists (physicians and non-physicians). POCUS has many applications, both in emergency/specialty tier 2–4 settings and non-emergency primary care settings (tier 1). In Uganda, rural non-physician clinicians use POCUS more often than imaging department-performed studies [39]. Task-shifting ultrasound of the breast is an appropriate alternative to mammography in Africa, considering the lack of infrastructure for mammography programs and the natural history of the disease locally [40]. POCUS for HIV and TB diagnosis is also well established [41].

Health systems should optimize recruitment and retention of qualified staff, especially those with advanced training and skills. On the other end of the tier-based system, barriers to task shifting include concerns about funding, licensing, and health worker concerns [24, 42]. Task-shifting also requires resources, training, and innovative strategies to be successful, and safe and its implementation must be contextual and accompanied by supervision and quality management [20, 43, 44].

## Infrastructure

Infrastructure includes a wide range of complex building blocks ranging from physical structural components to supporting services like transportation, energy, and computer bandwidth. Unlike other aspects of health care, access to reliable infrastructure for even the most basic imaging services is vital to sustainability and function. The two most rudimentary infrastructure components required for tier 1 basic imaging services include electricity and internet access—both of which are priorities included in UN SDGs [45]. Many hospitals and clinics in LMICs do not even have access to reliable electricity sources whereas the world bank states that broadband internet access is “not a luxury, but a basic necessity for economic and human development” [46].

**Fig. 5:**
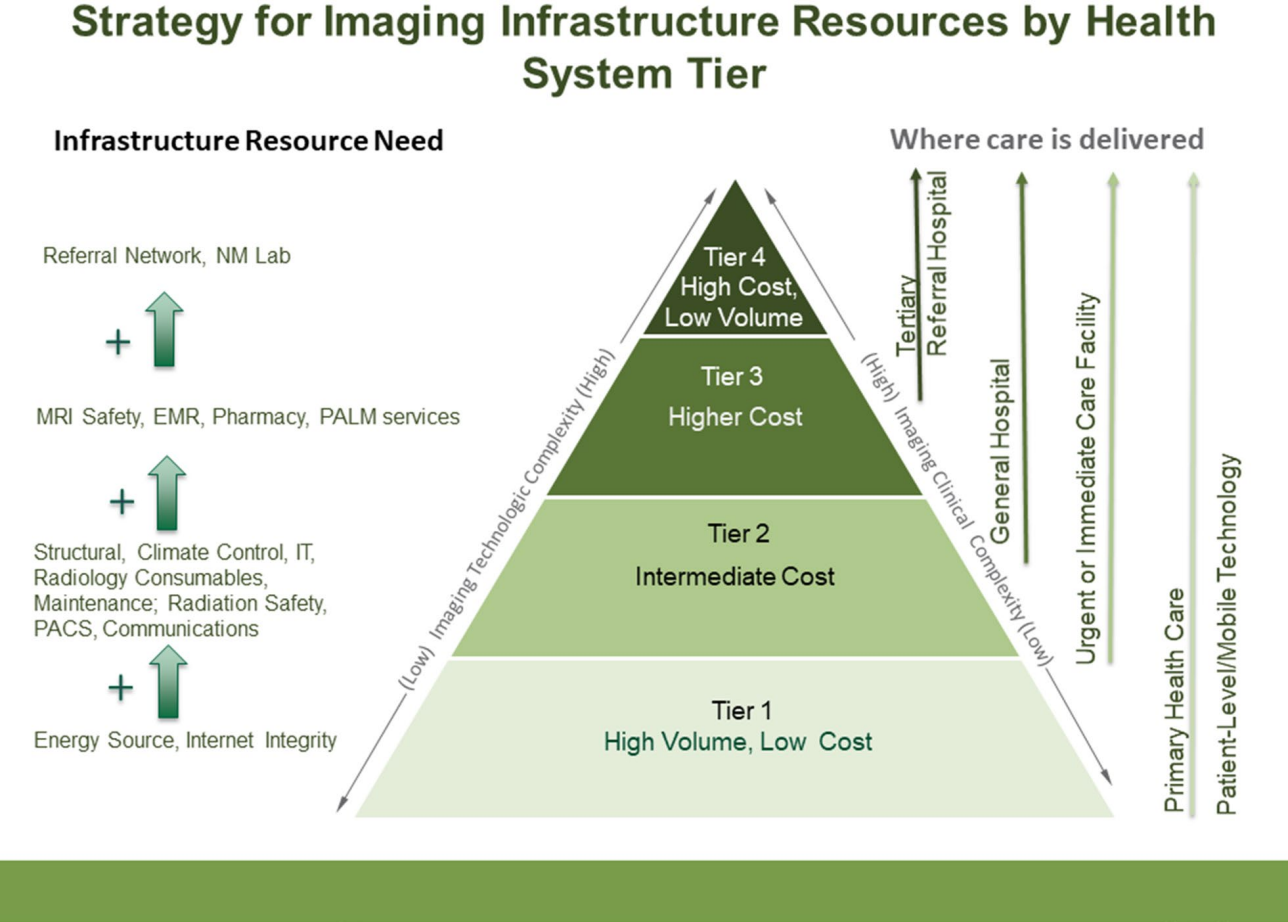
Recommended imaging infrastructure resource strategy by health system tier. Higher levels on the pyramid denote increasing complexity and requirement for more sophisticated and specialized infrastructure. Green arrows indicate added infrastructure required at the next highest tier (POCT = Point-of-care testing; IT = Information Technology; PACS = Picture Archiving and Communication system; MRI = Magnetic resonance imaging; EMR = electronic medical record; NM = nuclear medicine; PALM = pathology and laboratory medicine)

As the tiers increase in complexity of service, the infrastructure needs to increase significantly (Fig. 5). Building upon the basic energy source and internet integrity, several key infrastructure components are required to adequately sustain diagnostic ultrasound machines and X-ray units spanning tiers 1–4. These include radiation safety with structural shielding, imaging consumables, climate control and maintenance. If tele-consults or artificial intelligence (AI) platforms are being implemented as an imaging service for tier 1 or higher, a more robust IT system will need to be in place, including a picture archiving and communication system (PACS). Each machine will have an end-of-life (EOL) and end-of-service/support life (EOSL), which are both important to consider when procuring new or used products in order to ensure the optimal life of each machine. It is well known that donated imaging equipment and equipment procured without service contracts create challenges for LMICs and lead to inadequate equipment performance and use [18]. For referral healthcare centers in tiers 2–4 that provide multidisciplinary care, PACS, electronic medical records (EMR), and robust internet are essential.

Having insufficient access to transportation is a well-known rate-limiting step to providing many social and economic opportunities including the ability to procure many advanced medical devices, the maintenance of these devices, the procurement of imaging consumables and medications. To sustain the complex imaging devices requires easy access roads, nearby airplane landing strips, or boat docks given the importance of maintenance of these machines.

It is important to note, that as technology evolves, the requirements of infrastructure will also evolve. For example, ultrasound, X-ray machines, CT, and even MRI devices are becoming more portable and require less space and power, yet the ability to store images and provide remote quality care through cloud-based telecommunication systems is becoming standard in many parts of the world. Therefore, infrastructure needs will require continued updates and the capacity to evolve with new innovation.

## Quality and oversight

Unlike high-income countries (HICs), quality improvement programs are rarely legally mandated in LMICs. There is little published literature on imaging quality in LMICs, the majority focusing on quality control or radiation protection [46–48]. Very few papers report on quality improvement (QI) programs [47]. A survey of 23 leading hospitals in Ghana revealed complete absence of imaging quality assurance or quality improvement plans across the polled hospitals [49].

All LMICs can implement a quality improvement program. The QI vision should begin with the belief by political and healthcare leadership, that all people have a right to quality care so that this can translate into national healthcare quality improvement initiatives of diagnostic imaging services. A parallel step is to enact healthcare quality legislation featuring diagnostic services, and all-important buy-in from the stakeholders.

**Fig. 6:**
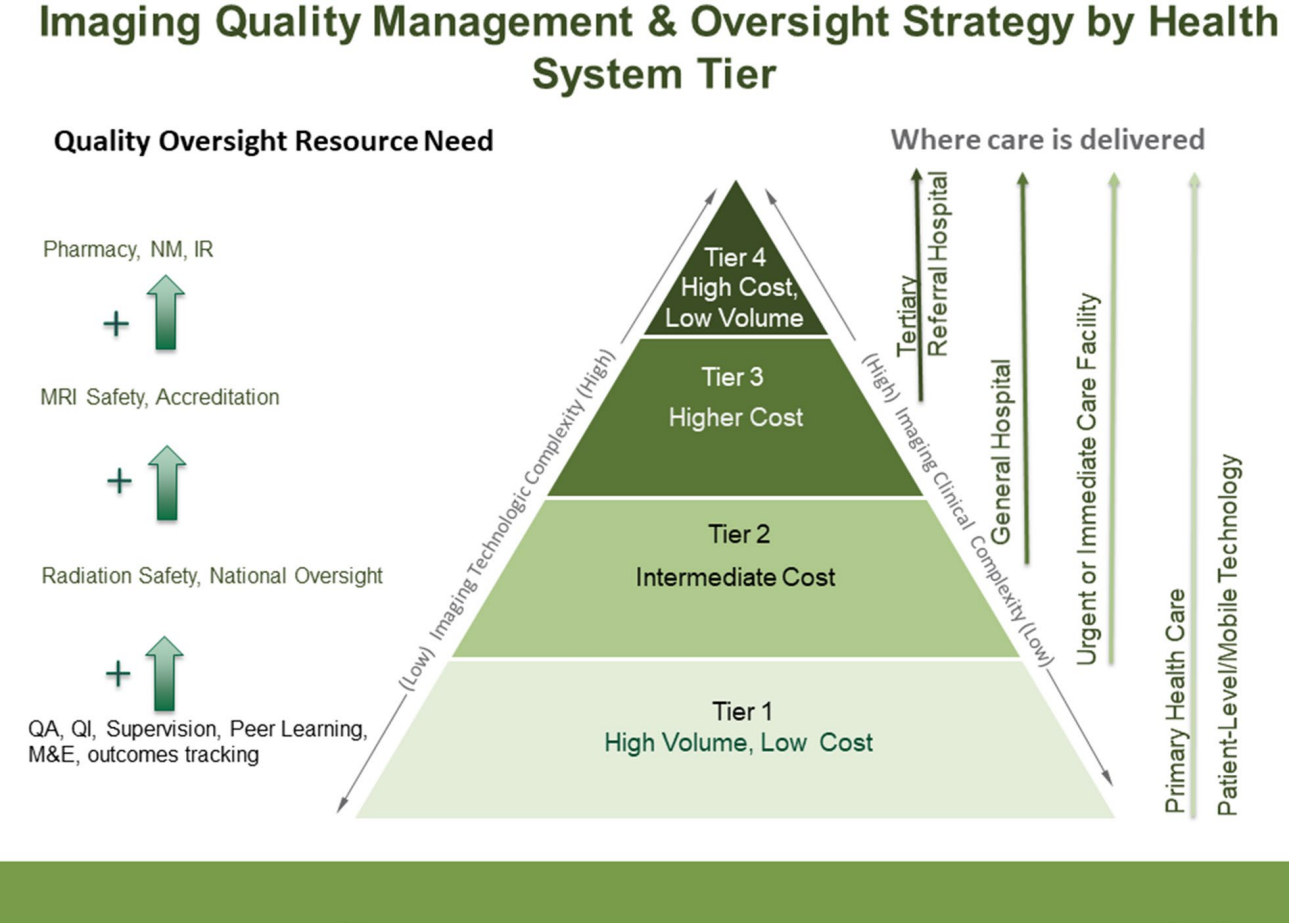
Recommended imaging quality management and oversight resource strategy by health system tier. Higher levels on the pyramid denote increasing complexity and additional requirement for more sophisticated, specialized quality management and oversight. Green arrows indicate added quality and oversight needed at the next highest tier (QA = Quality assurance; QI = quality improvement; M&E = monitoring and evaluation; MRI = magnetic resonance imaging; NM = nuclear medicine; IR = interventional radiology)

QI plans can be customized [50] by leadership and staff to fit all 4 Tiers of service (Fig. 6). Key elements of a QI plan are system orientation, and patient-centeredness, team building, education and training, data collection and analysis, infection control (especially hand hygiene), process checklists, diagnostic protocols, verification of the processes, and feedback. Data must be reported to the QI committee and up the chain of command, and QI plans should be revised according to lessons learned.

A QI plan for a tier 1 provided by non-radiologists performed ultrasound requires staff training and skills verification, Quality Control (QC) of acquired images, staying within accreditation standards, continuous skills enhancement, retention initiatives, and QI plan refinement, based on staff and patient feedback. For LMICs, the absence of skills verification is probably the weakest link in QI.

The focus of QI in Tier 2 is similar to Tier 1, but the presence of onsite radiologists allows for on-site supervision, real-time image QC, process verification, and data review, with real-time feedback to staff.

QI is pivotal to Tier 3, and critical for Tier 4 because these incorporate a full range of imaging modalities and procedures. Services, such as neuro-intervention and endovascular are highly risky, therefore skills verification, credentialing, and compliance with processes and protocols must be verified and recorded, for review. Departmental QI committees report to a quality officer, usually a senior radiologist. The committee analyzes QI data, especially complications and near misses, using statistical tools (e.g., Pareto chart, fish-bone diagram, or control chart). A commonly used methodology for affecting change is the Plan-Do-Study-Act (PDSA) cycle consisting of planning a change, trying it out on a small scale, observing the results, and acting on what is learned. All QI systems must adopt a methodology for change. Speaking up with ideas, and asking questions is to be encouraged at all tiers of imaging service.

## Conclusion

Strengthening national policy around tiered diagnostic imaging will support primary care and specialty services for the overall improved health of the population. Investment should be made into service delivery, technology, capacity, and capability strengthening of human resources, infrastructure development, and quality management systems. We have provided a novel framework for a tiered diagnostic imaging service, flexible enough to account for available resources in different contexts and settings. The COVID-19 pandemic proved that having a fluid, yet well-defined framework allows for maximum capacity utilization, even when the system is strained [51, 52]. In applying this framework, local radiologists should be empowered to inform decisions, support national policies, norms, and standards, and participate in cost-effectiveness studies that guide future policy.

## Data Availability

Not applicable.

## Abbreviations

ABI: Advanced breast imaging
B-IR: Basic interventional radiology
Complex IR: Complex interventional radiology
Complex R/S-US: Complex radiologist/specialist ultrasound
CT: Computed tomography
EOL: End-of-life (equipment)
EOSL: End-of service/support life (equipment)
FL: Fluoroscopy
MRI: Magnetic resonance imaging
NM: Nuclear medicine
NR/S-US: Non-radiologist/non-specialist ultrasound
NR: Non-radiologist
NR-U: Non-radiologist ultrasound
PACS: Picture archiving and communication system
PET: Positron emission tomography
POCUS: Point of care ultrasound
QC: Quality control
QI: Quality improvement
Subspecialized-IR: Subspecialized interventional radiology
